# Use of the extended feasible stability region for assessing stability of perturbed walking

**DOI:** 10.1038/s41598-020-79955-y

**Published:** 2021-01-13

**Authors:** Hosein Bahari, Juan Forero, Jeremy C. Hall, Jacqueline S. Hebert, Albert H. Vette, Hossein Rouhani

**Affiliations:** 1 Department of Mechanical Engineering, University of Alberta, 9211-116 Street NW, Edmonton, AB T6G 1H9 Canada; 2 Glenrose Rehabilitation Hospital, Alberta Health Services, 10230-111 Avenue NW, Edmonton, AB T5G 0B7 Canada; 3 Faculty of Medicine and Dentistry, Katz Group Centre, University of Alberta, Edmonton, AB T6G 2E1 Canada

**Keywords:** Biomedical engineering, Mechanical engineering, Geriatrics, Rehabilitation, Dynamical systems

## Abstract

Walking stability has been assessed through gait variability or existing biomechanical measures. However, such measures are unable to quantify the instantaneous risk of loss-of-balance as a function of gait parameters, body sway, and physiological and perturbation conditions. This study aimed to introduce and evaluate novel biomechanical measures for loss-of-balance under various perturbed walking conditions. We introduced the concept of ‘Extended Feasible Stability Region (ExFSR)’ that characterizes walking stability for the duration of an entire step. We proposed novel stability measures based on the proximity of the body’s centre of mass (COM) position and velocity to the ExFSR limits. We quantified perturbed walking of fifteen non-disabled individuals and three individuals with a disability, and calculated our proposed ExFSR-based measures. 17.2% (32.5%) and 26.3% (34.0%) of the measured trajectories of the COM position and velocity during low (high) perturbations went outside the ExFSR limits, for non-disabled and disabled individuals, respectively. Besides, our proposed measures significantly correlated with measures previously suggested in the literature to assess gait stability, indicating a similar trend in gait stability revealed by them. The ExFSR-based measures facilitate our understanding on the biomechanical mechanisms of loss-of-balance and can contribute to the development of strategies for balance assessment.

## Introduction

The risk of falling during daily life increases with aging^1^ and in the presence of chronic neuromuscular disorders^2^. Falls pose a noticeable threat to the growing population of elderly people^3^ as they can result in serious physical injuries^4^ or psychosocial complications due to self-imposed restrictions caused by fear of falling^5^. Quantifying the risk of falling contributes to designing prevention strategies and, thus, reducing the incidence of falling.

A considerable number of falls occurs during walking^6^. Stable walking can be defined as “gait that does not lead to falls despite perturbations”^7^. On the one hand, walking stability has been characterized using measures based on dynamic system stability^7^, such as maximum Lyapunov exponent^8^, long-range correlation^9^ and variability measures^10^. These measures assess the ability of the system to nullify the effects of small external perturbations. Yet, these measures based on dynamic system stability and variability have not been proposed to quantitatively characterize the effects of sudden external perturbation conditions (e.g., the base of support (BOS) motion, a trip or a slip) on the risk of loss-of-balance and the role of gait kinematics (e.g., lower limb joint angles, body sway, stride length or speed) to reduce this risk. Such a quantitative characterization can help training the individual to adjust the gait kinematics to prevent a loss-of-balance in response to each external perturbation, rather than the general assessment of one’s walking stability. On the other hand, walking stability can be characterized using measures derived from biomechanical models^11,12^. Such measures have the potential to account for the effects of external perturbations and effects of physiological (e.g., muscle strength) and environmental (e.g., slippery surface) conditions on the loss-of-balance. This is because the biomechanical models can take these perturbations and conditions into account in the solution of differential equations of motion and balance. Many of these biomechanical measures are based on the relative position and velocity of the body’s centre of mass (COM) with respect to its BOS (hereafter referred to as the COM state) at every instance of time. These measures obtain the limits of a feasible stability region (FSR) in which the COM state should lie to maintain dynamic balance during walking. Complex biomechanical models of the body represented as an inverted pendulum in combination with optimization processes have been proposed to obtain the FSR limits for standing and walking^13–16^.

Forward and backward loss-of-balance in response to external perturbations were studied in the past^17–21^. Previous studies observed that backward loss-of-balance could depend on the intensity of the external perturbations^22^. Yet, the previously introduced measures of stability based on biomechanical modeling did not characterize the loss-of-balance as a function of the intensity and shape of the external perturbation conditions. Indeed, the development of targeted balance training programs in interactive environments requires characterization of loss-of-balance due to various external perturbations in daily conditions, and as a function of dynamic body posture during walking. As such, these training programs could promote low-risk dynamic postures during perturbed walking to lower the risk of falling^20^. Interactive environments such as a Computer-Assisted Rehabilitation Environment (CAREN) have been developed to create various complex perturbation conditions and observe their effect on the loss-of-balance. There is currently no measure for walking stability that can quantify the effects of such complex perturbations on the loss-of-balance during continuous walking.

**Figure 1:**
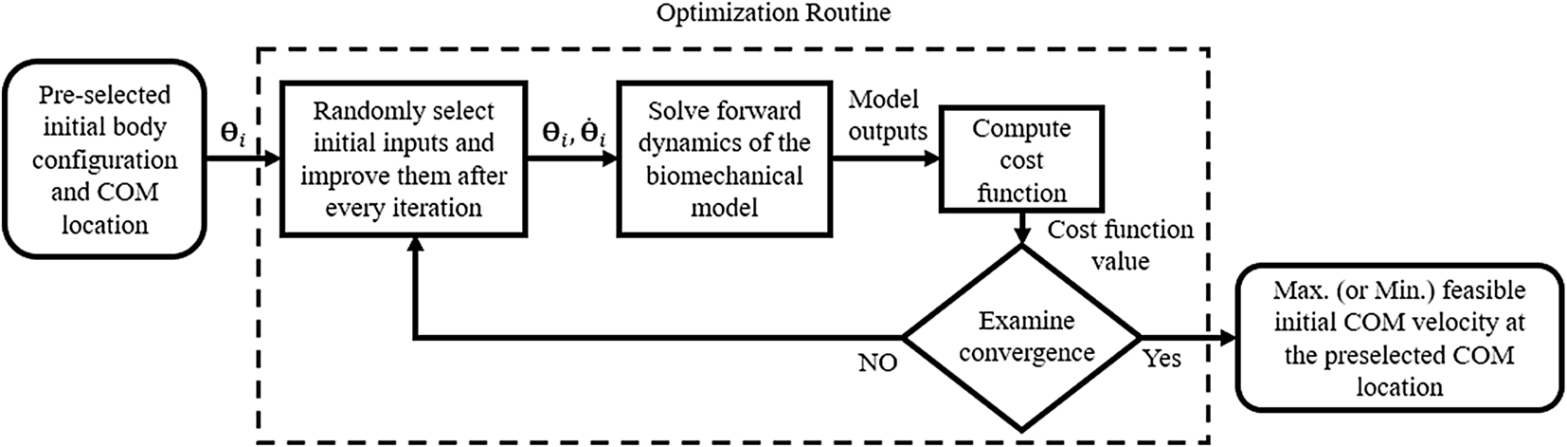
Illustration of optimization and simulation process used to obtain the FSR limits for perturbed walking conditions, based on the perturbation type and level. The output of the optimization process is the maximum and minimum feasible normalized COM velocity for every initial COM position^16,23^. External perturbations were in the form of vertical and horizontal displacements and sagittal rotations of the BOS. The FSR limits were obtained for various frequencies and amplitudes of perturbation (ranging from ± 1 cm to ± 6 cm in the vertical and horizontal directions and ± 0.6 degrees to ± 3 degrees in rotation).

We recently adopted a seven-segment bipedal model of human walking, proposed a methodology to obtain the FSR limits as a function of the amplitude and frequency of complex BOS perturbations in the sagittal plane, and validated it for balance at the toe-off instant^23^. This methodology obtained the FSR limits for a wide range of BOS perturbation that can be implemented in an interactive training environment such as the CAREN. In our previous work, for each initial position of the body COM, a range of initial COM velocities was found that would end the COM motion above the BOS, resulting in a stable posture. This range of initial COM velocities for each initial COM position defined the FSR limits. To obtain the FSR limits an optimization approach was employed to find the optimal torque, initial angular position and velocity of the joints in a seven-segment model of the body under various BOS perturbation circumstances and physiological constraints (Fig. 1). However, our previously obtained FSR was limited to the toe-off instant and not able to characterize the risk of both forward and backward loss-of-balance during the entire step duration. For example, the risk of forward loss-of-balance at the heel-strike instant could not be characterized using our previous study. In addition, although FSR limits were obtained, no previous study proposed and validated a ‘*gait stability measure*’ based on the FSR limits to characterize loss-of-balance as a function of the COM motion state, and a wide range of perturbation conditions during an entire step.

**Figure 2:**
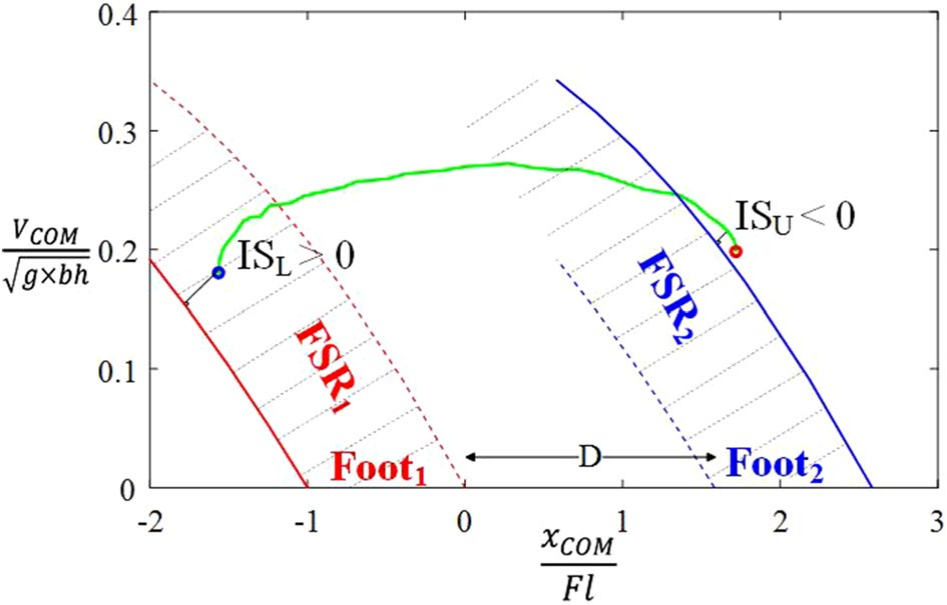
Extended Feasible Stability Region (ExFSR), along with the body’s COM motion states for the duration of an entire step. The ExFSR is the area between the solid red and blue lines that included FSR_1_ and FSR_2_ (shaded areas). The COM state trajectory is shown as a green line between Foot_2_’s toe-off instant (hollow blue circle) to Foot_1_’s toe-off instant (hollow red circle). The indexes of stability (*IS*_*L*_*(n)* and *IS*_*U*_*(n)*) illustrate how close the individual can be to backward and forward loss-of-balance, respectively, for the duration of a step. In the experiments, the distance between Foot_1_’s toe tip to Foot_2_’s heel (D) was measured using the reflective markers attached to each foot. The COM position and velocity values are referenced with respect to Foot_1_’s toe (stance foot during the swing phase before double support).

The primary objective of the present study is to extend our previously obtained FSR to an entire step duration and use it to define biomechanical stability measures in the sagittal plane during perturbed walking. To this end, in the present study, we extended the use of FSR to the duration of an entire step composed of a swing phase and the following double-support phase during continuous walking. We defined a new concept, Extended Feasible Stability Region (ExFSR), consisting of the FSR pertaining to both feet and space defined by the distance between them (Fig. 2). The secondary objective is to investigate the convergent validity of our proposed stability measures by showing their correlation with other previously reported stability measures using experimental data gathered during perturbed walking in a CAREN setup. Note that although previously reported stability measures were not able to quantify the risk of loss-of-balance as a function of the COM motion state and BOS perturbation conditions, they were still able to distinguish populations with high risk of falling. Thus, we hypothesize that the convergent validity of our proposed measures can be shown if they show a significant correlation with previously reported measures across populations, despite their different mathematical definition.

## Results

15 non-disabled individuals (body height: 1.79 ± 0.09 m and body mass: 79 ± 6 kg; mean ± standard deviation) and three individuals with a disability (body height: 1.78 ± 0.1 m; body mass: 76 ± 4 kg)—one with unilateral trans-femoral amputation, one with unilateral trans-tibial amputation, and one with unilateral upper limb amputation and sustained traumatic brain injury—participated in this study. They walked under a “low-perturbation” and “high-perturbation” walking conditions on a split-belt treadmill on a CAREN setup. For each step, we defined indexes of stability, i.e., *IS*_*L*_ and *IS*_*U*_, as the minimum distances between the lower and upper limit of ExFSR, respectively, and COM state trajectory. A negative value of *IS*_*L*_ (or *IS*_*U*_) indicates that the COM state is outside of the lower (or upper) limit of ExFSR resulting in a temporary loss-of-balance that needs to be recovered during the next step to prevent the incidence of falling. Therefore, a smaller value of *IS*_*L*_ or *IS*_*U*_ is associated with a higher risk of backward or forward loss-of-balance, respectively.

Our proposed measures (*IS*_*L,avg*_ , *IS*_*U,avg*_) based on *IS*_*L*_ and *IS*_*U*_ (described in Table 1) were calculated for each participant. In addition, inter-stride variability of gait cycle time and swing phase percentage (*GCT*_*MAD*_ , *GCT*_*nMAD* ,_ *SPP*_*MAD* ,_ and *SPP*_*nMAD*_) and the average of the minimum distance between the Extrapolated COM (XCOM) and the BOS boundaries (*b*_*min,avg*_) were also calculated to investigate the validity of our proposed measures.

**Table 1:** List of our proposed measures of stability and their associated parameters as well as previously introduced measures of stability.

Symbol	Definition
${IS}_{L,avg}$	Average of *IS*_*L*_*(n)*^a^ among all steps during one walking trial
${IS}_{U,avg}$	Average of *IS*_*U*_*(n)* among all steps during one walking trial
${BC}_{L}$	Percentage^b^ of steps in which *IS*_*L*_*(n)* is a negative value during one walking trial
${BC}_{U}$	Percentage of steps in which *IS*_*U*_*(n)* is a negative value during one walking trial
${b}_{min,avg}$	Average of all ${b}_{min}$ ^c^ values during one walking trial
${GCT}_{MAD}$	MAD^d^ among all GCT^e^ values during one walking trial
${GCT}_{nMAD}$	nMAD%^f^ among all GCT values during one walking trial
${SPP}_{MAD}$	MAD among all SPP^g^ values during one walking trial
${SPP}_{nMAD}$	nMAD% among all SPP values during one walking trial

Each parameter’s symbol and definition are presented.

^a^*n* is the step number index. *IS*_*L*_*(n)* (or *IS*_*U*_* (n)*) is the minimum distances between the lower (or upper) limit of ExFSR and COM state trajectory, during each step.

^b^Percentage refers to the fraction of steps (out of all steps of a walking trial) in which *IS(n)* had a negative value.

^c^*b*_*min*_ is the minimum distance between the XCOM and BOS boundaries during each step.

^d^*MAD* indicates the Median Absolute Deviation of parameter X among all steps of a walking trial: $MAD\left(X\right)=med\left(\left|X-med(X)\right|\right)$ (see^24^).

^e^*GCT* indicates the gait cycle time.

^f^*nMAD%* indicated the MAD normalized to the median value: $nMAD\%=100\times MAD/med$ (We introduced *nMAD%* as a robust alternative to the coefficient of variation).

^g^*SPP* indicates the swing phase percentage.

**Figure 3:**
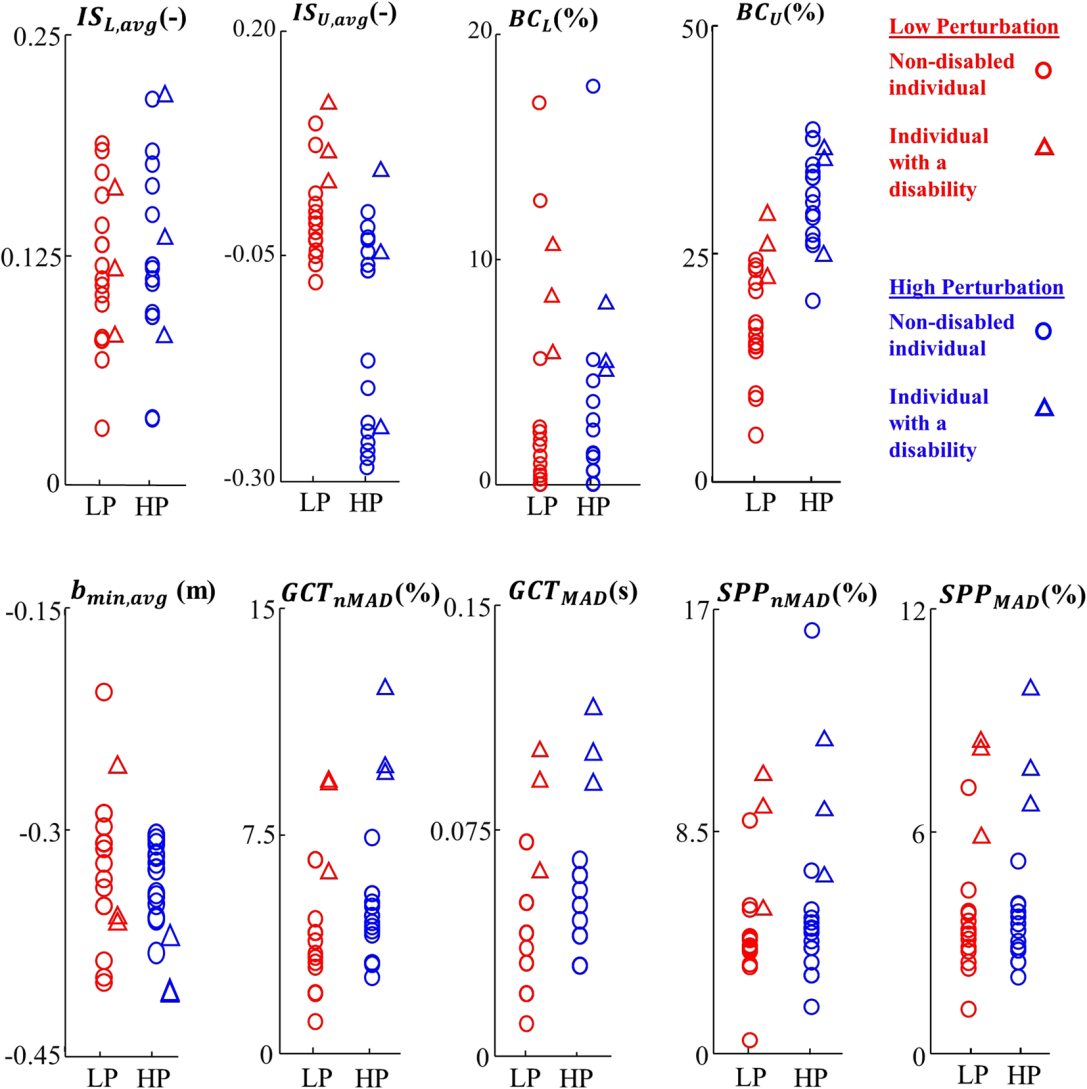
Stability measures obtained using experimental data. Our proposed measures (*IS*_*L,avg*_ and *IS*_*U,avg*_), *BC*_*L*_ and *BC*_*U*_, XCOM-based biomechanical measure (*b*_*min,avg*_), and variability measures (*GCT*_*nMAD*_, *GCT*_*MAD*_, *SPP*_*nMAD*_, and *SPP*_*MAD*_) are presented for non-disabled participants (circle) and participants with disability (triangle), for low-perturbation (*LP*, red) and high-perturbation (*HP*, blue) walking trials.

According to Fig. 3, *IS*_*U,avg*_ significantly decreased in high-perturbation condition compared to low-perturbation condition for non-disabled individuals (Wilcoxon sign-rank test’s p-value < 0.001). Since only three individuals with a disability participated in this study, we did not perform statistical analyses for this group and between the groups. Yet, the variability measures of stability (*GCT*_*MAD*_*, GCT*_*nMAD*_*, SPP*_*MAD*_, and *SPP*_*nMAD*_) tended to be larger in both perturbation conditions for individuals with a disability compared to non-disabled individuals (Fig. 3). However, individuals with a disability were able to improve their *IS*_*L,avg*_ and *IS*_*U,avg*_ compared to non-disabled individuals.

*BC*_*L*_ and *BC*_*U*_ quantified the percentage of instantaneous backward and forward loss-of-balance (without incidence of falling), respectively, reported by our stability measures. The average (among participants) of *BC*_*L*_ and *BC*_*U*_ were 2.7% (4.5%) and 16.8% (31.5%), respectively, in the low-perturbation (high-perturbation) condition for non-disabled participants. For the three participants with a disability, the averages of *BC*_*L*_ and *BC*_*U*_ were 9.5% (6.2%) and 25.7% (31.7%), respectively, in the low-perturbation (high-perturbation) condition, and they tended to be larger compared to those of non-disabled participants.

For both perturbation conditions, *IS*_*L,avg*_ and *IS*_*U,avg*_ significantly correlated with *b*_*min,avg*_. Unlike *IS*_*U,avg*_, the correlation coefficient between *IS*_*L,avg*_ and *b*_*min,avg*_ was negative (Table 2). *SPP*_*MAD*_ significantly correlated with *IS*_*L,*avg_ in both conditions and with *IS*_*U,*avg_ in the high-perturbation condition. Also, *SPP*_*nMAD*_ significantly correlated with *IS*_*L,*avg_ and *IS*_*U,*avg_ in the high-perturbation condition.

**Table 2:** The correlation coefficients between our proposed measures of stability and previously introduced biomechanical and variability measures.

		${b}_{min,avg}$	${GCT}_{nMAD}$	${GCT}_{MAD}$	${SPP}_{nMAD}$	${SPP}_{MAD}$
${IS}_{L,avg}$	$LP$	**− 0.65 (0.01)**	− 0.10 (0.72)	− 0.08 (0.77)	− 0.44 (0.06)	**− 0.54 (0.03)**
	$HP$	**− 0.66 (0.01)**	− 0.06 (0.84)	− 0.07(0.81)	**0.70 (0.00)**	**0.72 (0.00)**
${IS}_{U,avg}$	$LP$	**0.65 (0.01)**	− 0.03 (0.93)	− 0.12(0.68)	0.34 (0.21)	0.34 (0.21)
	$HP$	**0.57 (0.03)**	0.24 (0.39)	0.17(0.54)	**− 0.58 (0.02)**	**− 0.61 (0.02)**

In each cell, the correlation coefficient and the p-value for testing the hypothesis of no correlation (in parentheses) are presented. Significant correlations (p-value < 0.05) are bolded. *LP* and *HP* stand for low-perturbation and high-perturbation walking conditions.

## Discussion

In this study, we extended the use of FSR to the duration of an entire step composed of a swing phase and the following double-support phase during continuous walking, by defining a new concept, ExFSR (see Fig. 2). For a COM state trajectory starting from the toe-off instant of one foot (Foot_2_) and ending with the toe-off instant of the next foot (Foot_1_), if the trajectory lies within the ExFSR limits, no further balance recovery actions (e.g., taking extra steps forward or backward, or upper body counter-motion) are required. However, if the trajectory passes these limits, at least a temporary loss-of-balance will occur. We defined *IS*_*L,avg*_ and *IS*_*U,avg*_, as our proposed measure of stability (see Table 1), to characterize the loss-of-balance as a function of body sway (that determines COM states), step length, foot length and perturbation conditions.

We used the FSR limits obtained based on a biomechanical model developed in our previous work^23^ and in this study, introduced the ExFSR limits and proposed novel stability measures capable of characterizing the biomechanical risk of loss-of-balance during an entire step, as a function of the BOS perturbation profile. To evaluate our proposed measures, we defined *BC*_*L*_ and *BC*_*U*_ as the percentage of steps in which the COM states were outside of the lower and upper limits of ExFSR. The averages of *BC*_*L*_ and *BC*_*U*_ were 2.7% (4.5%) and 16.8% (31.5%) for non-disabled participants, and 9.5% (6.2%) and 25.7% (31.7%) for participants with a disability during the low (high) perturbation condition. The observed values of *BC*_*L*_ and *BC*_*U*_, especially the small values observed in low-perturbation condition for non-disabled participants indicate the extent of validity of our obtained ExFSR limits and proposed stability measures. Notably, observing COM states in some instants outside of the ExFSR limits cannot necessarily question the validity of our proposed stability measures. COM states outside of the FSR limits without an incidence of falling were also observed in previous works^16,23^. Indeed, a temporary loss-of-balance is usually recovered with various strategies, but their frequency could be an indicator of an increased risk of falling. In this line, the obtained *BC*_*L*_ and *BC*_*U*_ tended to increase for participants with a disability, and *BC*_*U*_ increased with the perturbation level (Fig. 3).

We also observed different changes of backward and forward loss-of-balance in high-perturbation walking conditions. *IS*_*L,avg*_ and *BC*_*L*_ for non-disabled individuals did not show any significant change between low-perturbation and high-perturbation conditions. In contrast, *IS*_*U,avg*_ decreased and *BC*_*U*_ increased (p-value < 0.001) for non-disabled individuals in high-perturbation conditions compared to low-perturbation conditions. In addition, unlike *IS*_*U,avg*_, the correlation coefficient between *IS*_*L,avg*_ and *b*_*min,avg*_ was negative. This may indicate that when the risk of loss-of-balance increased due to large external perturbations, these individuals pushed the COM state away from the lower limit of ExFSR towards the more interior regions of ExFSR and closer to its upper limit to avoid a backward loss-of-balance. This can be justified since it is easier to recover forward loss-of-balance by taking extra steps forward. In other words, the neuromuscular control system tends to show more flexibility towards a forward loss-of-balance and chooses a larger distance to the lower limit of ExFSR. In this line, previous studies also showed that different neural circuits process the postural response to the forward and backward perturbations^21^. Hence, both *IS*_*L,avg*_ and *IS*_*U,avg*_ should be used separately for dynamic balance assessment.

The XCOM-based margin of stability (*b*_*min,avg*_) uses a simplified model of the body and an approximation of the actual FSR. Also, it does not provide details on how the loss-of-balance is a function of the intensity or shape of the BOS perturbation^25,26^. As such, our proposed measures of stability (*IS*_*L,avg*_ and *IS*_*U,avg*_) as well as *BC*_*L*_ and *BC*_*U*_ have further modelling capacities and do not obtain identical results with *b*_*min,avg*_ (Fig. 3). Nevertheless, our proposed stability measures significantly correlated with the *b*_*min,avg*_ (i.e., correlation correction: significantly different with zero). In other words, they show a similar trend in increasing or decreasing gait stability in the presence of low or high perturbation or disability, although our proposed measures provide more detailed information on the risk of loss-of-balance. The observed correlation indicated the convergent validity of our proposed stability measures against *b*_*min,avg*_ as an established measure of dynamic stability in the literature^22,27–29^. Yet, the correlation coefficient between our proposed measures and *b*_*min,avg*_ was moderate and not high (close to 1). Hence, *b*_*min,avg*_ cannot be a substitute for our proposed measures.

In order to better duplicate a close to natural gait, the treadmill speed was chosen to be self-paced to adapt to each individual and its speed constantly changed for each participant during each trial. By duplicating a close to natural gait for all participants and minimizing the impact of a fixed speed of the treadmill, we expect that the observed inter-participant differences among the non-disabled group indicated the individual’s adaptability to the perturbation. Note that although altering speed results in a different number of steps during each trial among participants, our proposed measures were either defined as an average value among all steps (*IS*_*L*_ and *IS*_*U*_) or as a percentage of all the steps (*BC*_*L*_ and *BC*_*U*_) during each trial. This means that, despite having a different number of steps for each participant, the outcome of our measures can still be compared among different individuals.

A range of disability was chosen to reflect their effects and the effect of BOS and COM manipulation strategies to cope with their consequences on the outcome of our proposed measures of stability. While an upper limb amputation mainly affects COM position, our study participant also suffered from a brain injury that further affected his walking. It is difficult to make conclusions about the exact effects of each disability on the outcome of our measures since only one participant was involved with each disability. We were only able to illustrate that our proposed stability measure can account for a diverse range of disabilities that affect the individual’s physiological and biomechanical abilities to maintain balance. The large inter-participant variability among the three participants with a disability was due to the different impacts of their disability on walking balance. Yet, the balance outcome of one individual cannot be generalized to all individuals with trans-tibial or trans-femoral amputation or those with a brain injury.

Our proposed measures are obtained with respect to ExFSR boundaries and depend on COM motion states, body height and BOS (that is a function of the step length). Therefore, our proposed measures depend on gait strategies utilized by an individual. Our results showed that individuals with a disability were partially able to adjust their gait strategy to keep their COM states away from ExFSR boundaries. Although they showed comparable *IS*_*L,avg*_ and *IS*_*U,avg*_ with the non-disabled individuals, the individuals with a disability tended to have a larger *BC*_*L*_ and *BC*_*U*_ meaning that they tended to have more instances of temporary loss-of-balance during perturbed walking. It was also previously observed that individuals with unilateral trans-tibial amputation adjust their gait and COM states to improve their gait stability^29^. Under similar perturbation conditions, we expect that our proposed measures of stability will show a larger difference between disabled and non-disabled individuals if they are forced to walk at the same pace.

In dynamical systems, an increase in variability of the system’s behaviour can be associated with instability and chaos in the system^10^. Variability measures have noticeable popularity among researchers in the assessment of walking stability due to their simple calculation and understandable concept^30,31^. Nevertheless, increased variability in complex dynamical systems is not always indicative of chaos, but can also arise from the system characteristics and existence of multiple degrees of freedom in the system^10^. In low-perturbation walking trials, inter-stride variability of gait parameters can be due to a combination of both the system’s internal dynamics and the external perturbation^7^. Since these two effects could hardly be separated from each other, variability measures would be less meaningful in low-perturbation walking trials^32^. In high-perturbation walking trials, the effect of the external perturbation on the dynamical system is amplified and, thus, variability measures are more susceptible to change. In this line, we observed correlations between the SPP variability and our proposed measures, particularly in the high-perturbation condition. In contrast to the *SPP* variability, the *GCT* variability did not correlate with our proposed measures. This could be due to the effects of the varying speed of the treadmill on the inter-stride variability of *GCT*. In general, variability measures might not be reliable for stability assessment of perturbed walking conditions as it is not possible to separate the effects of the external perturbation from the internal dynamics of the system. In addition, the non-stationary nature of gait parameter sequences due to the external perturbation may cause overestimations of the variability measure.

The calculation of our proposed measures required measurements of only the body’s COM trajectory and BOS motion since the employed FSR limits are already formulated as a function of various complex BOS perturbation in our previous work^23^. The COM trajectory and BOS motion can be easily measured using various motion capture technologies, which facilitates clinical balance assessment and enables several clinical applications of our proposed approach to loss-of-balance characterization. Our experimental results included both non-disabled participants and participants with a disability to show the potential of our proposed stability measures to be utilized for both groups under various perturbation conditions. Yet, the practical challenges of this methodology should be further investigated in larger populations with pathological gait.

## Study limitations

Our study has a number of limitations. First, our proposed measures of stability are limited to sagittal plane movements of the body and BOS. Notably, the ExFSR limits are specific to the type of BOS perturbations. Similar to our previous study^23^, to obtain the ExFSR limits, we only considered dominant BOS perturbations in the sagittal plane and characterized biomechanical mechanisms of the loss-of-balance in the sagittal plane. Perturbations in the sagittal plane were applied in several previous studies to investigate mechanisms of backward and forward loss-of-balance^18,20,22,33,34^. Yet, gait is a three-dimensional motion, and movements in the frontal plane affect the stability in the sagittal plane. A future perspective of our study is to determine the ExFSR limits in the frontal plane and for other types of BOS perturbations.

Second, our biomechanical model considered the head-arms-trunk as a rigid body segment and was not capable of accounting for upper body perturbations or the effect of upper limb motion on balance recovery^26^. Although the contribution of upper limb motion was neglected in several previous studies^12,16^, it can modify the limits of loss-of-balance and the risk of falling. A further segmented model of head-arms-trunk, able to account for the upper body perturbations as external forces in the dynamic optimization routine, is required to analyze the effects of upper body perturbations on gait. Also, similar to the previous studies^16,23^, we did not use active pendulum length because, first, our participants did not crouch and walked upright and thus we neglected the deformation of the head-arms-trunk segment in our modelling; second, the trochanteric length was considered as a fixed value throughout the optimization whereas active pendulum length alters based on the body configuration. Considering the variable active pendulum length would prevent the use of our modelling approach and presenting the normalized FSR limits.

Third, in addition to biomechanical mechanisms and measured of loss-of-balance discussed here, individual-specific physiological (e.g., muscle conditions), cognitive conditions, and balance training would affect the risk of loss-of-balance and falling^35^. The marginal value for *IS*_*L*_ (or *IS*_*U*_) at which the actual loss-of-balance leads to incidence of falling depends on physiological and cognitive characteristics of an individual. Although our obtained ExFSR limits would contribute to developing strategies for prediction and prevention of falling, future work should characterize the individual-specific thresholds of our proposed measures for which loss-of-balance transitions to falling occur based on individual-specific measurements.

## Conclusions

This study introduced a set of stability measures based on the concept of ExFSR and using a previously developed seven-segment biomechanical model of the human body in the sagittal plane. These measures are able to characterize biomechanical mechanisms of loss-of-balance during walking, as a function of BOS perturbation, gait parameters (e.g., step length), and body motion pattern (e.g., COM state). Compared to the previously introduced FSR-based stability measures, our proposed measures are able to characterize the risk of both forward and backward loss-of-balance during an entire step. Our proposed measures use the outcome of our previous study^23^ to relate the risk of loss-of-balance during continuous walking to a range of complex perturbation profiles. As such, these measures can contribute to our understanding on human balance control for biped walking, and the strategies for balance assessment in interactive training environments such as the CAREN.

## Methods

### Modelling loss-of-balance

#### ExFSR

In our previous study^23^, we adopted and revised a seven-segment bipedal model of a walking human introduced by Yang et al.^16^ and obtained the FSR at the toe-off instant of the swing foot (Foot_2_) where the BOS was the standing foot (Foot_1_) in contact with the ground (Fig. 2). The lower and upper limits of the FSR as a function of the BOS perturbation amplitude and frequency are reported in Tables 1 and 2 of this reference^23^ and are used in the present work. The lower limit of FSR defined the backward loss-of-balance as a need for stepping backward to prevent falling. The upper limit of FSR defined the forward loss-of-balance as the inability to maintain balance by terminating gait where the anterior foot is located and without taking further steps forward. During continuous walking, the body COM state voluntarily leaves this FSR (FSR_1_) during the swing phase period, which would not necessarily result in loss-of-balance. This is because the BOS expands to the area under and between both feet as soon as Foot_2_ touches the ground in front of Foot_1_ at the beginning of the double-support phase (i.e., the heel-strike instant of Foot_2_).

During the double-support phase, until the succeeding toe-off instant of Foot_1_, forward loss-of-balance does not occur before the COM state passes the upper limit of the succeeding FSR (FSR_2_) in which the anterior foot (Foot_2_) determines the BOS. In the present paper, to expand the definition of FSR to assess the stability of consecutive steps, we define the ExFSR (Extended FSR) as the region including the FSRs for all instants within an entire step (from the toe-off instant of Foot_2_ to the toe-off instant of Foot_1_). Given that each step is composed of a swing phase and a succeeding double-support phase, the ExFSR is the COM state-space between the lower limit of FSR_1_ and the upper limit of FSR_2_ (Fig. 2). Note that during the double-support phase, FSR_1_ and FSR_2_ are separated by the distance between the toe’s tip of Foot_1_ and the heel of Foot_2_. We assumed that FSR_1_ and FSR_2_ are identical and can be obtained based on the frequency and amplitude of the external perturbations according to ^23^. During walking, the COM, BOS and, thus, ExFSR progress step by step.

#### Index of stability

We introduce the ‘Index of Stability’, i.e., *IS*_*L*_*(n) and IS*_*U*_*(n)*, to characterize the risk of backward and forward loss-of-balance, respectively, during one isolated step during gait (*n* is the step index). *IS*_*L*_*(n)* (or *IS*_*U*_*(n)*) are defined as follows (Fig. 2):

(i) The shortest distance, a positive value, from the trajectory of the COM state to the lower (or upper) limit of the ExFSR, if the COM state lies inside the ExFSR near its lower (or upper) limit.
(ii) The longest distance, a negative value, from the trajectory of the COM state to the lower (or upper) limit of the ExFSR, if the COM state lies outside the lower (or upper) limit of the ExFSR.

*IS(n)* (*i.e., IS*_*L*_*(n)* or *IS*_*U*_*(n)*) depends on both the COM position and velocity, and illustrates how close the individual can be to loss-of-balance for the duration of a step. When *IS(n)* is a positive value, the smaller the *IS(n),* the more probable the loss-of-balance. A negative *IS(n)* is indicative of temporary loss-of-balance. Based on the physiological condition of the walker and the value of the negative *IS(n)*, the temporary loss-of-balance can either be recovered or lead to an incidence of falling.

#### Index of balance challenge

We also defined the ‘Index of Balance Challenge’, *BC*_*L*_ and *BC*_*U*_, as the percentage of steps (out of all steps) during a walking trial, in which *IS*_*L*_*(n)* and *IS*_*U*_*(n)*, respectively, was negative. As such, *BC*_*L*_ and *BC*_*U*_ are indicators of challenge in maintaining backward and forward balance, respectively, during a perturbed walking trial.

### Experimental protocol

To validate our proposed measures of stability against existing stability measures, we conducted a set of experiments using a CAREN. The same experimental data were used as in^23^. 15 non-disabled individuals and three individuals with a disability participated in this study. All participants gave informed consent to perform the experiments approved by the research ethics board of the University of Alberta (protocol number: Pro00066076), and all experiments were performed in accordance with relevant guidelines and regulations. Each participant walked on a platform-mounted treadmill in 60-m trials under a “low-perturbation” and a “high-perturbation” walking condition. During both low- and high-perturbation trials, the BOS perturbations occurred in the form of continuous vertical and horizontal displacements and sagittal rotations of the CAREN platform. The dominant frequencies and amplitudes of the low (high) perturbation were 1 Hz (3 Hz) and 1 cm (6 cm) in the vertical and horizontal directions, respectively, and of 1 Hz (3 Hz) and 0.6 deg (3 deg) in the rotational direction, respectively. The perturbation profiles were randomized for every participant and every trial and did not have an established pattern. All three types of perturbations were applied during the same trial by super-positioning these three types. The participants were notified of perturbation occurrence 3 s prior to each the start of perturbation through a visual message appearing on the CAREN screen. Treadmill speed was adapted to the participant’s preferred walking speed to mimic the natural walking condition. The perturbation trials were performed after an initial familiarization where the participants were asked to walk on the treadmill with their preferred pace to adapt to the self-paced condition. Four reflective markers were used to track the motion of the platform. We tracked the BOS motion using four markers mounted on a rigid plate attached to each foot that obtained the trajectory of the heels and toes. Four markers were mounted on another plate attached over the sacrum, and were used to obtain the body COM motion based on the location of the COM with respect to the sacrum suggested by Yang and Pai ^36^. Normalized COM position and velocity with respect to the BOS at each instant were used to calculate the COM state trajectory. The ExFSR for the duration of consecutive steps were obtained using (i) the corresponding FSRs for each perturbation profile computed using the equations proposed by Bahari et al. ^23^ and (ii) the distance between the toe tip of the posterior foot (Foot_1_) and heel of the anterior foot (Foot_2_) during the double-support phase (Fig. 2).

### Data analysis

*IS*_*L*_*(n)* and *IS*_*U*_*(n)* were calculated for both lower and upper limits of the ExFSR and presented as an average among all steps in a walking trial (*IS*_*L,avg*_ and *IS*_*U,avg*_). *BC*_*L*_ and *BC*_*U*_ were also calculated for each walking trial. We compared *IS*_*L,avg*_, *IS*_*U,avg*_, *BC*_*L*_ and *BC*_*U*_ in the non-disabled group between the high-perturbation and low-perturbation conditions using Wilcoxon signed-rank test. In addition, for each step in each gait trial, we calculated the following stability measures to investigate potential correlations between those and our proposed measures. The definition and symbol used for each stability measure are given in Table 1.

#### Margin of stability based on the XCOM

Hof et al.^25,26^ used an inverted pendulum model of the human body and suggested that, for dynamic stability of walking and standing, the body XCOM should remain within the BOS limits:

1 $$XCOM={P}_{COM}+\frac{{V}_{COM}}{{\omega }_{0}} , {\omega }_{0}= \sqrt{\frac{g}{l}}$$

where ${P}_{COM}$ and ${V}_{COM}$ are the body’s COM position and velocity, *g* is the gravitational acceleration and *l* is the equivalent trochanteric length of each participant. They defined the margin of stability (*b*) as the shortest distance between the XCOM and the BOS boundaries, and calculated it for every instant. The most unstable moment is when the value of *b* is minimum (*b*_*min*_) within a step. We chose *b*_*min*_ because it is the most widely used biomechanical stability measure in the literature^7,25,37^. We calculated *b*_*min*_ for each step and considered the average of *b*_*min*_ among all steps (*b*_*min,avg*_) as a measure of stability during a walking trial.

#### Variability measures

The variability of gait parameters such as *GCT* and *SPP* (defined in Table 1) has been introduced as an indicator of gait stability and risk of falling^38,39^. To characterize the inter-stride variability of GCT and SPP, we calculated robust measures, i.e., MAD ^24^ and nMAD% (Table 1), among all gait cycles of each walking trial.

#### Correlation analysis

To investigate if our proposed measures of stability show, in general, a similar trend of gait stability with variability measures and margin of stability (*b*_*min,avg*_), despite their different mathematical definitions, we calculated (i) all of the above measures for the two perturbed walking trials of each participant; and (ii) the Spearman’s correlation coefficient between these measures among participants.
